# Adiponectin C1Q and Collagen Domain Containing rs266729, Cyclin-Dependent Kinase Inhibitor 2A and 2B rs10811661, and Signal Sequence Receptor Subunit 1 rs9505118 Polymorphisms and Their Association with Gestational Diabetes Mellitus: A Case-Control Study in a Romanian Population

**DOI:** 10.3390/ijms26041654

**Published:** 2025-02-14

**Authors:** Mihai Muntean, Claudiu Mărginean, Elena Silvia Bernad, Claudia Bănescu, Victoria Nyulas, Irina Elena Muntean, Vladut Săsăran

**Affiliations:** 1 Department of Obstetrics and Gynecology 2, George Emil Palade University of Medicine, Pharmacy, Science, and Technology of Târgu Mureș, 540142 Târgu Mureș, Romania; mihai.muntean@umfst.ro (M.M.); vladut.sasaran@umfst.ro (V.S.); 2 Department of Obstetrics and Gynecology, Faculty of Medicine, “Victor Babes” University of Medicine and Pharmacy, 300041 Timisoara, Romania; bernad.elena@umft.ro; 3 Clinic of Obstetrics and Gynecology, “Pius Brinzeu” County Clinical Emergency Hospital, 300723 Timisoara, Romania; 4 Center for Laparoscopy, Laparoscopic Surgery and In Vitro Fertilization, “Victor Babes” University of Medicine and Pharmacy, 300041 Timisoara, Romania; 5 Genetics Laboratory, Center for Advanced Medical and Pharmaceutical Research, George Emil Palade University of Medicine, Pharmacy, Science, and Technology of Târgu Mureș, 540142 Târgu Mureș, Romania; claudia.banescu@umfst.ro; 6 Departament of Informatics and Medical Biostatistics, George Emil Palade University of Medicine, Pharmacy, Science, and Technology of Târgu Mureș, 540142 Târgu Mureș, Romania; victoria.rus@umfst.ro; 7 Algcocalm SRL, 540360 Târgu Mureș, Romania; irinagauca@yahoo.com

**Keywords:** SNPs, gestational diabetes mellitus, ADIPOQ, CDKN2A/2B, SSR1, gene

## Abstract

Gestational diabetes mellitus (GDM) and type 2 diabetes mellitus (T2DM) are public health concerns worldwide. These two diseases share the same pathophysiological and genetic similarities. This study aimed to investigate the T2DM known single nucleotide polymorphisms (SNPs) of the adiponectin C1Q and collagen domain containing (*ADIPOQ*), cyclin-dependent kinase inhibitor 2A and 2B (*CDKN2A/2B*), and signal sequence receptor subunit 1 (*SSR1*) genes in a cohort of Romanian GDM pregnant women and perinatal outcomes. DNA was isolated from the peripheral blood of 213 pregnant women with *(n* = 71) or without (*n* = 142) GDM. Afterward, *ADIPOQ* (rs266729), *CDKN2A/2B* (rs10811661), and *SSR1* (rs9505118) gene polymorphisms were genotyped using TaqMan Real-Time PCR analysis. Women with GDM had a higher pre-pregnancy body mass index (BMI) (*p* < 0.0001), higher BMI (*p* < 0.0001), higher insulin resistance homeostatic model assessment (IR-HOMA) (*p* = 0.0002), higher insulin levels (*p* = 0.003), and lower adiponectin levels (*p* = 0.004) at birth compared to pregnant women with normoglycemia. GDM pregnant women had gestational hypertension (GH) more frequently during pregnancy (*p* < 0.0001), perineal lacerations more frequently during vaginal birth (*p* = 0.03), and more macrosomic newborns (*p* < 0.0001) than pregnant women from the control group. We did not find an association under any model (allelic, genotypic, dominant, or recessive) of *ADIPOQ* rs266729, *CDKN2A/2B* rs10811661, and *SSR1* rs9505118 polymorphisms and GDM. In correlation analysis, we found a weak positive correlation (r = 0.24) between the dominant model GG + CG vs. CC of rs266729 and labor induction failure. In the dominant model TT vs. CC + CT of rs10811661, we found a weak negative correlation between this model and perineal lacerations. Our results suggest that the *ADIPOQ* rs266729, the *CDKN2A/2B* rs10811661, and the *SSR1* rs9505118 gene polymorphisms are not associated with GDM in a cohort of Romanian pregnant women.

## 1. Introduction

The World Health Organization (WHO) report from 2022 showed that the incidence of overweight adults and obesity in adults over 20 years has increased in Europe in recent years [1]. Unfortunately, childhood and adolescent overweight and obesity incidences have also increased, occurring in up to 21% of American adolescents [2,3]. Obesity in adolescents is associated with an increased risk of insulin resistance, type 2 diabetes mellitus (T2DM), hypertension, cardiovascular diseases, musculoskeletal problems, sleep disorders, depression, and cancer later in life [2,4].

T2DM and GDM are two forms of diabetes mellitus (DM).

T2DM is defined as a value of glycosylated hemoglobin A1c (HbA1c) ≥ 6.5%, fasting plasma glucose ≥ 126 mg/dL, or 2 h plasma glucose ≥ 126 mg/dL during an oral glucose tolerance test, or a patient with symptoms of hyperglycemia or hyperglycemic crisis and a random glucose ≥ 200 mg/dL [5].

GDM is defined as diabetes diagnosed in the second or third trimester of pregnancy that was not diagnosed before gestation as T2DM or another type of diabetes (type 1 diabetes mellitus) throughout the first trimester of pregnancy [5]. These two forms of DM have almost the same risk factors, such as a high glycemic index diet, overweight, obesity, impaired glucose tolerance, physical inactivity [6,7,8], family history of DM, and specific ethnicity (non-Hispanic Black women, Pima Indians) [6,7,8]. GDM and T2DM also share the same metabolic abnormalities, such as increased insulin resistance, decreased insulin secretion, and altered levels of adipokines like adiponectin [6,8,9,10], and consequently, 40% of women with a history of GDM will develop T2DM in the next 15 years after a pregnancy affected by GDM [7].

In addition to environmental and lifestyle factors, the genetic component of T2DM occurrence is highlighted by the 72% heritability in monozygotic twin pairs [11]. There is increasing evidence that GDM has both epigenetic and genetic components, with certain genetic variants linked to GDM also being prevalent in T2DM [12,13,14]. DNA methylation of genes like insulin-like growth factor-2 (*IGF-2*) related to insulin and glucose metabolism is one of the epigenetic mechanisms involved in the development of GDM [14,15]. The polymorphisms of the insulin receptor gene (*INSR*) [16], and of transcription factor 7-like 2 (*TCF7L2*) [17] are examples of gene polymorphisms associated with GDM.

Some genome-wide association studies (GWAS) showed that rs266729 single nucleotide polymorphisms (SNP) in the promoter of the *ADIPOQ* gene [18,19] are associated with GDM and adiponectin serum levels in GDM patients, suggesting that genetics play a role in circulating adiponectin in pregnant women with GDM [19]. The *ADIPOQ* gene is located on chromosome 3q27 and consists of three exons and two introns. SNPs in the *ADIPOQ* gene are also associated with T2DM and type 1 DM (T1DM) [20].

Rs10811661 polymorphism of the gene *CDKN2A/2B*, localized on chromosome 9p21.3, is associated with altered beta cell function, impaired insulin release, and impaired glucose tolerance (IGT) [21]. *CDKN2A/2B* encodes two kinase inhibitors essential for beta cell function. GWASs show that rs10811661 polymorphism was associated with GDM and T2DM risk in Chinese populations [22,23]. Conversely, some studies show that a higher number of C alleles of rs10811661 were protective against GDM [24].

Another SNP associated with GDM [25] and T2DM [26] is rs9505118 of the *SSR1* gene, on chromosome 6p24.3. The *SSR1* gene is involved in regulating fasting insulin and fasting glucose levels [27].

Based on the above findings, we hypothesized that SNP rs266729 in the *ADIPOQ* gene, rs10811661 in the *CDKN2A/2B* gene, and rs9505118 in the *SSR1* gene are associated with GDM and perinatal outcomes.

This study focused on comparing the single nucleotide polymorphisms (SNPs) rs266729 in the *ADIPOQ* gene, rs10811661 in the *CDKN2A/2B* gene, and rs9505118 in the *SSR1* gene among a cohort of Romanian GDM patients versus healthy pregnant women. Additionally, it explored the perinatal outcomes and the relationship between these polymorphisms and perinatal outcomes.

## 2. Results

### 2.1. Maternal Demographic, Anthropometric, and Biochemical Parameters at Birth

Table 1 presents the demographic, anthropometric, and biochemical parameters of the pregnant women included in the study.

We found that GDM patients had a significantly higher incidence of a heredo-collateral history of T2DM (*p* < 0.0001) and a lower gestational age at delivery than the control group (*p* = 0.001).

In the case of anthropometric parameters, GDM patients were overweight before pregnancy and became obese grade 1 at birth. They had a greater pre-pregnancy BMI (*p* < 0.0001), a greater BMI at birth (*p* < 0.0001), and a lower GWG (*p* = 0.004) than the control group. The values of MUAC and TST were also greater than those of the controls (*p* < 0.0001 and *p* = 0.001, respectively). CRP values were not different between the groups (*p* = 0.68). Regarding glucose homeostasis parameters and insulin resistance parameters at birth, the GDM group had significantly higher values than the control group (HgbA1c (*p* < 0.0001), insulin (*p* = 0.003), and C-peptide (*p* = 0.008)). Serum adiponectin values at birth in the GDM group were significantly lower than in the control group (*p* = 0.004). However, in the GDM group, there were no differences in adiponectin values at birth between different rs266729 genotypes (CC vs. CG *p* = 0.9, CC vs. GG *p* = 0.8).

Table 2 shows newborn anthropometric characteristics. Newborns from GDM were heavier than those from the control group (*p* = 0.01). The MUAC values of newborns from GDM mothers were greater than those of newborns from the control group mothers (*p* = 0.03). There was no difference between groups regarding the 5 min APGAR score or newborn gender.

In a multivariate logistic regression analysis, we found that a higher pre-pregnancy BMI independently predicted an increased risk of GDM (Table 3), with an OR of 1.247 for pre-pregnancy BMI.

### 2.2. Maternal-Fetal Outcomes

Maternal-fetal outcomes are shown in Table 4. Pregnant women with GDM from our cohort had GH more frequently during pregnancy (*p* < 0.0001), experienced perineal lacerations during vaginal birth (*p* = 0.03), and delivered macrosomic newborns (*p* < 0.0001) more often than healthy pregnant women from the control group. There were no differences in the incidence of cesarean section rates between groups (*p* = 0.76).

### 2.3. Association Between Maternal Studied Gene Polymorphisms and the Risk of GDM

Table 5 shows the frequencies and distribution of alleles and genotypes in the rs266729, rs10811661, and rs9505118 polymorphisms in GDM and control women. Under any model (the allele model, the genotypic model, the codominant model, the dominant model, and the recessive model), we did not find significant differences in allele and genotype frequencies of all studied SNPs between the GDM and control groups (*p* > 0.05).

### 2.4. Correlations Between rs266729, rs10811661, and rs9505118 Polymorphisms and Maternal-Fetal Outcomes

In correlation analysis, we found a weak positive correlation (r = 0.24) between the dominant model GG+CG vs. CC of rs266729 and labor induction failure. In the dominant model TT vs. CC+CT of rs10811661, we found a weak negative correlation between the rs10811661 SNP and perineal lacerations. Table 6 shows correlations between the studied SNPs and maternal-fetal outcomes.

## 3. Discussion

In our study, we found that women from the GDM group had a higher incidence of a heredo-collateral history of T2DM and delivered at a lower gestational age compared to the control group. They exhibited greater adiposity, insulin resistance, and lower adiponectin levels at birth than the control group. Pregnant women with GDM in our cohort experienced GH more frequently during pregnancy, suffered perineal lacerations during vaginal births, and delivered macrosomic newborns more often than those in the control group. We did not observe significant differences in allele and genotype frequencies of all examined SNPs between the GDM and control groups. In correlation analysis, we identified a weak positive correlation between the dominant model GG+CG versus CC of rs266729 and labor induction failure. In the dominant model TT versus CC+CT of rs10811661, we found a weak negative correlation between the rs10811661 SNP and perineal lacerations.

Concerning the higher incidence of heredo-collateral history of T2DM from our cohort, this aligns with the findings of McIntyre et al.’s [8] findings. Additionally, Monod et al. [28] found that pregnant women with both parents diagnosed with T2DM exhibited worse glucometabolic profiles at the end of the first trimester and were more frequently diagnosed with GDM during pregnancy, indicating the influence of genetic and environmental factors on GDM occurrence. We collected data regarding the heredo-collateral history of T2DM without distinguishing between first- or second-degree relatives affected by T2DM or between pregnant women with affected mothers, fathers, or both.

In the case of greater adiposity, Zhu et al. [29], Dias et al. [30], and Tangjittipokin et al. [19] also found that GDM pregnant women have higher pre-pregnancy BMI and greater BMI at birth compared to the control pregnant group. In obese pregnant women, weight gain during gestation leads to an increase in visceral and subcutaneous adipose tissue. This results in visceral adipose tissue dysfunction and the release of inflammatory cytokines (tumor necrosis factor—TNF-α, interleukin-6 (IL-6), and CRP), which can alter hepatic glucose production. Consequently, this leads to increased glucose output from the liver, reduced glucose uptake in muscle tissue, and heightened insulin resistance [31,32,33].

During a healthy pregnancy, increased insulin resistance is counterbalanced by an upregulation of beta cell function, which maintains normoglycemia. We found that our GDM pregnant women had higher levels of insulin and IR-HOMA at birth compared to the control group. Ellerbrock et al. [34] and Pan et al. [35] also discovered that in obese pregnant women with GDM, insulin resistance plays a more significant role in the occurrence of GDM than beta cell dysfunction.

Adipose tissue has endocrine functions. Adiponectin is a peptide hormone secreted by adipose tissue. It is found in multiple multimeric complexes and has insulin-sensitizing, anti-atherogenic, and anti-inflammatory properties [36]. Low levels of adiponectin throughout all stages of gestation are linked to a higher risk of metabolic dysfunction during pregnancy and an increased incidence of GDM [37]. Our findings indicate that serum adiponectin levels at birth were significantly lower in the GDM group compared to the control group, which is consistent with our results from a previous study [9]. One explanation for lower adiponectin levels in obese patients comes from Kim et al. [38]’s work. They demonstrated that epigenetic changes, such as DNA methylation of the adiponectin R2 promoter, inhibit adiponectin expression and worsen metabolic disturbances in obese individuals. Moyce et al. [39] showed in a study on mice that adiponectin deficiency led to increased hepatic lipid accumulation during pregnancy; consequently, this deficiency contributed to glucose intolerance, dysregulated gluconeogenesis, and hyperglycemia.

Regarding the higher incidence of GH in our GDM group compared to the control group, Parrettini et al. [40] could provide one explanation. They emphasized in their review the crucial role of maternal obesity and excessive weight gain in fostering a pro-inflammatory state and endothelial dysfunction within the fetoplacental unit. This condition can lead to insulin resistance and an exaggerated vascular response to vasoconstrictors, which are common pathogenic factors for GDM and GH. Carpenter et al. [41] suggest that GDM is associated with an overexpressed innate immune response related to vascular dysfunction and disease.

Concerning the higher macrosomic newborns in our GDM group, Parettini et al. [40] revealed in their work that secondary to systemic low-grade inflammation, insulin resistance, increased maternal insulinemia and hyperglycemia, increased placental volume, and impaired placental genes that regulate cell cycle parameters, lipid metabolism, and mitochondrial activity, fetuses of GDM mothers had increased growth and adiposity, leading to macrosomia. Li et al. [42] also concluded in their study that increased insulin resistance during pregnancy was associated with macrosomia in Chinese women with GDM rather than beta cell dysfunction.

Our findings regarding higher perineal tears in GDM patients during vaginal births align with those of Fabricius et al. [43]. They conducted a systematic review and meta-analysis, revealing that primiparous women with GDM face a higher risk of obstetric anal sphincter injury compared to those without the condition. Von Theobald et al. [44] also a strong association between GDM and grades 3–4 of deep perineal trauma in cases of operative delivery. Conversely, Strand-Holm et al. [45] did not find a higher risk of lower genital tract tears among women diagnosed with diabetes (type 1 Diabetes Mellitus, T2DM, and GDM) compared to those without diabetes. It is important to mention that in our unit, there is routine use of episiotomy during the vaginal births of primiparous women and selective use of episiotomy for multiparous women. Our higher rate of perineal tears in the GDM group may be related to a higher incidence of newborn macrosomia, and possibly to a deficiency of perineal protection techniques.

Previous studies have shown that GDM and T2DM share similar metabolic abnormalities, including insulin resistance and β-cell dysfunction [6,7,8]. Additionally, some genetic variants associated with GDM are also common to T2DM [12,13], highlighting the genetic component in the emergence of these diseases.

The *ADIPOQ* gene encodes adiponectin, and SNPs of the *ADIPOQ* gene, such as rs266729, have been associated with the occurrence of GDM [20]. We found no differences between the groups regarding the allele and genotype distribution of rs266729 in the *ADIPOQ* gene. Pawlik et al. [18] found that the G allele of rs 266729 was an independent predictor of an increased risk of GDM. Beltcheva et al. [46] reported that the C allele of rs 266729 is associated with GDM, likely influencing the transcription process of adiponectin. Consistent with our results, Zhu et al. [29], and Dias et al. [30] found no associations between the rs266729 polymorphism and GDM. The differences in these results may arise from the varying G allele frequency across different populations and the fact that GDM is a multifactorial disease in which diet and physical activity play significant roles [47].

Hribal et al. [21] found that the rs10811661 polymorphism in the *CDKN2A/2B* gene is associated with altered beta cell function, impaired insulin release, and IGT. In their meta-analysis of multiethnic studies, Guo et al. [22] demonstrated that carriers of the T allele of the *CDKN2A/2B* rs10811661 have a moderate risk of developing GDM. Additionally, Li et al. [23] found that the rs10811661 polymorphism was significantly associated with the risk of T2DM. On the contrary, Tarnowski et al. [24] demonstrated that a higher number of C alleles of the rs10811661 SNP offer protection against GDM in a cohort of 411 pregnant women, both with and without GDM. Conversely, we did not find an association between the *CDKN2A/2B* rs10811661 polymorphism and GDM. Noury et al. [48] reported similar findings in a cohort of 98 Egyptian pregnant women, regardless of their GDM status.

Scott et al. [27] found that the *SSR1* gene regulates fasting insulin and fasting glucose by influencing preproinsulin translocation across the endoplasmic reticulum membrane for proinsulin biosynthesis. Data available for the rs9505118 polymorphism’s role in T2DM and GDM are limited and conflicting. Kasuga et al. [25], in a cohort of 299 Japanese pregnant women with and without GDM, found that *SSR1* rs9505118, *ADIPOQ* rs266729, and *CDKN2A/2B* rs10811661 are associated with the development of GDM. In a GWAS, Mahajan et al. [26] found that the *SSR1* rs9505118 polymorphism, among other polymorphisms, had a role in T2DM susceptibility. Contrary to this, Matsuba et al. [49] in 7620 Japanese patients with and without T2DM found that the *SSR1* rs9505118 polymorphism is not associated with T2DM. Also, we did not find an association between the *SSR1* rs9505118 polymorphism and GDM. The discrepancy in results among these studies may be attributed to variations in the frequencies of this polymorphism across different ethnicities, the number of participants included in each study, and the influence of environmental factors on the polymorphism expression.

In correlation analysis, we found a weak positive correlation between the dominant model GG + CG versus CC of rs266729 and labor induction failure. Several factors increase the likelihood of failed labor induction in obese patients, including lower cervical dilation at admission, nulliparity, fetal weight above 4000 g, and pregnancies complicated by GH and GDM, which raise the necessity for labor induction before term. The proposed pathophysiologic mechanism for failed labor induction in obese patients includes decreased myometrial contractile function. This is caused by the inhibition of intramyometrial calcium influx by higher levels of leptin and cholesterol in obese pregnant women, which antagonizes the effect of oxytocin [50]. Since we did not assess the leptin and cholesterol levels of the pregnant women included in the study, nor their relation to the rs266729 polymorphism, and given the low r-value (r = 0.24), we cannot conclude that the statistical significance of the association between the dominant model GG + CG versus CC of rs266729 and failed labor induction is clinically relevant for these patients. This warrants further studies in the future.

Regarding protective factors of perineal tears, obesity [44,51], modified Ritgen maneuver [52], perineal massage, the application of warm compresses during the second stage of labor [53], and side-lying position for birth [54] are protective against perineal tears during vaginal birth. In the dominant model TT versus CC + CT of rs10811661, we found a weak negative correlation between the rs10811661 SNP and perineal lacerations. More extensive prospective studies are needed to verify whether these genotypes influence the incidence of perineal tears during vaginal birth.

To the best of our knowledge, this is the first study to investigate the association among the *ADIPOQ* gene rs266729 polymorphism, the *CDKN2A/2B* gene rs10811661 polymorphism, and the *SSR1* gene rs9505118 polymorphism, and the development of GDM in a Romanian population.

We acknowledge that our study has several limitations. First, the number of our participants was a relatively small sample size for testing the associations of these SNPs with the disease phenotype. Second, we did not assess the patients’ diets and physical activity, which can significantly influence GDM occurrence, and we also did not evaluate the patients’ lipid profiles, which could affect GDM risk. Third, all participants in this study were recruited from a secondary maternity hospital in Târgu Mureș and thus may not be representative of pregnant Romanian women in Romania. Fourth, we collected data regarding the heredo-collateral history of T2DM without differentiating between first- or second-degree relatives affected by T2DM or between pregnant women with affected mothers, fathers, or both.

What are the implications of these findings for clinical practice and further research? Our results align with existing literature regarding the higher risk of gestational hypertension, macrosomia, and perineal tears during vaginal births in women with GDM. The SNPs we studied should not be used as markers for an increased risk of developing GDM.

Further studies are necessary to investigate the connection between diet, physical activity, adipokines, these SNPs, and GDM. Larger prospective studies are essential to clarify whether the *ADIPOQ* gene rs266729 and *CDKN2A/2B* gene rs10811661 genotypes are associated with perinatal adverse outcomes in GDM patients.

## 4. Materials and Methods

### 4.1. Study Design

The University of Medicine, Pharmacy, Science, and Technology “G. E. Palade” of Târgu-Mures Ethics Committee has authorized this study (decision number 1557/2022) following the principles of the Declaration of Helsinki (1964).

### 4.2. Description of Study Area and Duration of Study

This prospective case–control study was conducted between 1 February 2022, and 31 August 2024, in the Obstetrics–Gynecology Clinic 2 unit of County Hospital Mureș in Târgu Mureș, Romania.

### 4.3. Inclusion and Exclusion Criteria

The inclusion criteria were singleton pregnancy, diagnosis of GDM at 24–28 weeks of pregnancy, Romanian ethnicity, age above 18 years, and delivery at the Obstetrics and Gynecology Clinic 2 Târgu Mureș. The exclusion criteria were patients with T1DM or T2DM diagnosed before pregnancy, GDM diagnosis before 24 weeks of pregnancy, pregnancies with chromosomal anomalies or fetal malformations, cases of intrauterine fetal death, chronic infections, autoimmune and inflammatory diseases, neoplastic diseases, and those who lacked informed consent.

Before enrollment in the study, written informed consent was obtained from all pregnant women.

After applying the inclusion and exclusion criteria during the time mentioned above, we consecutively included 213 pregnant women in the study, divided into two groups based on the oral glucose tolerance test (OGTT) results: 71 with GDM and 142 healthy pregnant women as a control group. Structured questionnaires were used to obtain demographics (maternal age, gestation, parity, and first-degree family history of T2DM) and medical and reproductive history.

In all cases, the gestational age was determined using the date of the last menstrual period and a first-trimester ultrasound.

### 4.4. Diagnosis of GDM

For the diagnosis of GDM, we used The International Association of Diabetes and Pregnancy Study Groups (IADPSG) criteria [55]. One or more abnormal glucose values above ≥92 mg/dL (≥5.2 mmol/L) fasting, 1 h ≥ 180 mg/dL (≥10 mmol/L), or 2 h ≥ 153 mg/dL (≥ 8.5 mmol/L) after 75 g glucose ingestion were used for diagnosis.

Patients with GDM were instructed to participate in moderate exercise for 30 min per day, adhere to nutritional therapy (1600–1800 kcal per day with 35–40% carbohydrates), and track their glycemic levels by conducting three daily checks of fasting and postprandial glucose for two weeks as a part of our local protocol. The target glucose levels were set at fasting below 95 mg/dL and postprandial levels under 120 mg/dL, measured two hours after eating [56]. A diabetologist prescribed insulin therapy at 0.7–1.0 units/kg of body weight per day for women who did not achieve glycemic control through exercise and diet. Pregnant women continued to monitor their glucose levels until birth under the supervision of the diabetologist. All pregnant women had appointments every two weeks, or more frequently if necessary, until delivery as part of their routine prenatal care.

### 4.5. Anthropometric Measurements

We performed a series of maternal anthropometric measurements at admission to the hospital before birth on all pregnant women included in the study, including weight, height, mid-upper arm circumference (MUAC), tricipital skinfold thickness (TST), body mass index (BMI), and total weight gain during pregnancy. We used the patient’s pre-pregnancy weight, which was reported at the first prenatal visit, to calculate her pre-pregnancy BMI.

The patient’s height was measured in centimeters without shoes using a wall-mounted tape measure. The obtained value was estimated to the nearest 1 mm.

We used a Beurer PS digital scale (Beurer GmbH, Ulm, Germany) to assess the patient’s weight (kg), deducting 0.5 kg for the clothing.

The BMI was calculated by dividing the patient’s weight by the square of the height (kg/m^2^).

All newborns were measured for weight, length, MUAC, and TST during the first hour after birth.

The newborns’ weight was measured using the U-Grow electronic baby scale, U001-BS (Guangzhou Berrcom Medical Device Co., Ltd., Guangzhou, China), and their length was measured using an inextensible tape measure.

Using an inextensible tape measure, the MUAC was measured halfway between the acromion and olecranon of the posterior left upper arm. We used a Harpenden Skinfold Caliper (Baty International, West Sussex, UK) for TST measurement, calibrated to the nearest 0.2 mm. The measurements were conducted at the same location where the MUAC measurement was performed. Two measurements were obtained, and the average was computed and noted [57].

Information about newborn gender and 5 min APGAR scores was obtained from medical records.

### 4.6. Biochemical Analyses

We collected maternal blood samples upon hospital admission during the prepartum period. Shortly after collection, we assessed them for C-reactive protein (CRP), glycosylated hemoglobin A1c (HbA1c), insulinemia, C peptide, insulin resistance homeostatic model assessment (IR HOMA), and adiponectin levels.

HbA1c and CRP values were determined via turbidimetry, while insulinemia and C-peptide were measured by chemiluminescence with the Atellica Solution CH 930 device (Siemens Healthcare GmbH, Forchheim, Germany).

The formula used to estimate IR HOMA was [(fasting insulin (mU/L) × 209 fasting glucose (mmol/L)]/22.5 [58].

For adiponectin assessment, the blood samples were left in a serum separator tube at room temperature for 30 min to allow the serum to clot. The blood samples were centrifuged at 6000 rev/min for 4 min at room temperature. The serum was then separated and stored at −20 °C until assayed.

An automated enzyme immunoassay analyzer (DYNEX DSX Automated ELISA System, DYNEX Technologies Inc., Chantilly, VA, USA) was used to assess adiponectin levels using the Human Total Adiponectin/ACRP30 ELISA kits (PDRP 300, R&D Systems, Bio-techne, Minneapolis, MN, USA), adhering to the manufacturer’s protocol. The intra-assay coefficient of variation for adiponectin was <4.8% and the inter-assay coefficient of variation was <7.0%. The manufacturer states that the sensitivity of the assays for adiponectin is 0.246 ng/mL.

### 4.7. Genotyping Analysis

We collected maternal blood for genotyping and biochemical analysis simultaneously. Blood samples were collected in tubes containing ethylenediaminetetraacetic acid (EDTA) and stored at −20 °C until assayed. DNA was extracted using a PureLink™ kit (Invitrogen, Life Technologies Corp, Carlsbad, CA, USA).

We genotyped all samples by using the TaqMan genotyping methodology, TaqMan™ Fast Advanced Master Mix (ThermoScientific LSG, Waltham, MA, USA), and specific TaqMan^®^ pre-designed TaqMan^®^ SNP Genotyping Assays (Applied Biosystems, Foster City, CA, USA) to discriminate the *ADIPOQ* rs266729 (C_2412786_10), *CDKN2A/2B* rs10811661 (C_31288917_10), and *SSR 1* rs9505118 (C_1945197_10).

Genotyping was performed on the 7500 Fast DX Real-Time polymerase chain reaction (PCR) system (Applied Biosystems, Foster City, CA, USA).

### 4.8. Maternal and Neonatal Complications

We recorded the gestational age at birth, the mode of delivery, the anthropometric measurements of the mother and newborn described above, and adverse events during pregnancy (premature birth and gestational hypertension) and at birth (abdominal delivery, failed induction of labor (IOL), perineal lacerations, and macrosomia).

We defined maternal and neonatal complications as follows:

Spontaneous or medically indicated birth at less than 37 completed weeks of gestation, according to ICD-10 definitions (O60), was defined as premature birth.

A systolic blood pressure reading of 140 mm Hg or higher, or a diastolic blood pressure reading of 90 mm Hg or higher, or both, on two separate occasions at least 4 h apart after 20 weeks of pregnancy in a woman who previously had normal blood pressure was defined as gestational hypertension (GH) [59]. We initiated antihypertensive therapy with nifedipine at a persistent systolic blood pressure of 160 mmHg or more, diastolic blood pressure of 110 mmHg or more, or both [59].

Failed IOL is defined as not entering the active phase of labor after 24 h of prostaglandin administration or after 12 h of oxytocin infusion [60].

Perineal lacerations are classified according to the anatomical structures involved, from first-degree lacerations where only perineal skin is involved to fourth-degree lacerations where are involved anal sphincter complex and anal epithelium [61].

A newborn weight of ≥4000 g at birth was used to diagnose macrosomia [62].

### 4.9. Statistical Analysis

Statistical analyses were performed by using GraphPad Prism version 9.0 (GraphPad Software, Boston, MA, USA). Continuous variables were expressed as mean ± standard deviation and median (IQR). Categorical variables were represented as percentages. Student's *t*-test was utilized for normally distributed continuous variables, while the Mann–Whitney test was used for non-normally distributed data. The chi-square test for categorical variables assessed clinical characteristics between subjects with GDM and the control group. We performed a Spearman correlation analysis to identify significant correlations between polymorphisms and perinatal outcomes. Furthermore, we applied logistic regression analysis to identify potential predictors for GDM. A two-sided *p* < 0.05 was considered statistically significant.

We conducted a priori power analysis with the program G Power Version 3.1.9.6 from Faul et al. [63] using data from Beltcheva et al. [46]. Based on these data, we estimated a medium effect size of 0.4, assuming a two-tailed *t*-test with at least 80% power and alpha = 0.05. The total number of 217 patients will be the minimum required to sample for sufficient power, *n* = 145 in the control group and *n* = 72 in the GDM group. We assumed that 213 patients divided into 142 control patients and 71 GDM patients (2:1 ratio) would be enough for our study to have sufficient power.

## 5. Conclusions

Based on our results, we suggest that the ADIPOQ gene rs266729 polymorphism, the CDKN2A/2B gene rs10811661 polymorphism, and the SSR1 gene rs9505118 polymorphism are not associated with GDM in a cohort of Romanian pregnant women. Women in the GDM group had lower adiponectin levels at birth and experienced higher rates of gestational hypertension, perineal lacerations during vaginal delivery, and macrosomic newborns compared to those in the control group. Pre-pregnancy BMI acts as an independent predictor of an increased risk of GDM.

## Figures and Tables

**Table 1:** Demographic, anthropometric, and biochemical parameters of GDM and control cases at birth.

Parameters	GDM Group (*n* = 71)	Control Group (*n* = 142)	*p*-Value
Maternal age at delivery, Median (IQR)	33.0 (31.0–34.0)	31.0 (30.0–32.0)	0.051
Heredo-colateral history of T2DM, %	25 (35.2%)	15 (10.6%)	<0.0001
Gestation, Median (IQR)	2.0 (1.0–3.0)	2.0 (1.0–3.0)	0.2
Parity, Median (IQR)	2.0 (1.0–4.0)	1.0 (1.0–4.0)	0.2
Gestational age at delivery, weeks, Median (IQR)	38.6 (38.2–39.3)	39.2 (38.5–39.5)	0.001
Pre-pregnancy BMI, Kg/m^2^, Median (IQR)	27.58 (25.7–29.0)	22.1 (21.7–22.76)	<0.0001
GWG, Mean (SD)	12.7 ± 7.13	15.3 ± 5.49	0.004
BMI at birth, Kg/m^2^, Mean (SD)	33.1 ± 5.52	28.5 ± 3.8	<0.0001
MUAC, cm, Median (IQR)	31.5 (28.4–33.9)	28.0 (26.2–41.2)	<0.0001
TST, mm, Median (IQR)	22.2 (18.8–25.8)	19.6 (14.8–24.2)	0.001
CRP, mg/dL, Median (IQR)	0.76 (0.4–1.18)	0.62 (0.3–1.0)	0.68
HgbA1c, %, Median (IQR)	5.6 (5.4–6.1)	5.4 (5.2–5.5)	<0.0001
IR-HOMA, Median (IQR)	3.23 (2.0–4.2)	2.3 (1.6–3.4)	0.0002
Insulin, mUI/L, Median (IQR)	14.8 (10.1–20.8)	11.7 (8.4–16.3)	0.003
C-peptide, Median (IQR)	3.2 (2.4–3.9)	2.7 (2.0–3.4)	0.008
Adiponectin, ng/mL, Median (IQR)	6020 (4346–7306)	7131 (5272–8724)	0.004

Note: Data are presented as medians (standard deviation or interquartile range), counts, and percentages. T2DM = type 2 diabetes mellitus; BMI = body mass index; GWG = gestational weight gain; IQR = interquartile range, MUAC = mid-upper arm circumference; TST = tricipital skinfold thickness; CRP = C reactive protein; HbA1c = glycosylated hemoglobin; IR HOMA = insulin resistance homeostatic model assessment; SD = standard deviation.

**Table 2:** Newborn anthropometric characteristics.

Newborn Characteristics	GDM Group (*n* = 71)	Control Group (*n* = 142)	*p*-Value
Weight, g, Median	3470 (3170–3850)	3350 (3108–3603)	0.01
APGAR 5 min			
≥7	71 (100%)	142 (100%)	N/A
<7	0	0	N/A
Newborn gender, n, % Female, Male	38 (53.5%) 33 (46.5%)	68 (47.9%) 74 (52.1%)	0.47
MUAC, mm, Mean, (SD)	11.26 ± 1.08	10.9 ± 0.89	0.03
TST, mm, Mean (SD)	6.12 ± 1.45	5.67 ± 1.29	0.051

Note: Data are presented as medians (standard deviation or interquartile range), counts, and percentages. IQR = interquartile range; MUAC = mid-upper arm circumference; TST = tricipital skinfold thickness; SD = standard deviation.

**Table 3:** Multivariate logistic regression analysis assessing predictors of higher hazard of GDM.

Parameters	OR (95% CI)	*p*-Value
Maternal age	1.043 (0.973–1.118)	0.2
Pre-pregnancy BMI	1.247 (1.151–1.352)	0.0001
Parity (≥2)	1.057 (0.646–1.730)	0.8

Note: BMI = body mass index.

**Table 4:** Maternal-fetal outcomes.

Parameters	GDM Group (*n =* 71)	Control Group (*n* = 142)	*p*-Value
Gestational hypertension, n, %	12 (16.9%)	3 (2.1%)	<0.0001
Preterm birth (<37 weeks), n, %	5 (7.0%)	8 (5.6%)	0.4
Failure of labor induction, n, %	3 (4.2%)	2 (1.4%)	0.2
Cesarean section, n, %	45 (63.38%)	93 (67.39%)	0.76
Perineal lacerations, n, %	8 (11.3%)	5 (3.5%)	0.03
Macrosomia (≥4000 g), n, %	15 (21.1%)	5 (3.5%)	<0.0001

Note: data are presented as counts and percentages.

**Table 5:** Comparison of maternal genotype frequencies between GDM cases and control group.

Parameters	GDM Group (*n* = 71) %		Control Group (*n* = 142) %		*p*-Value
**rs 266729**					
**Alele**					
**C**	109	76.7%	215	75.7%	0.9
**G**	33	23.2%	69	24.2%	
**Genotype**					
**CC**	41	57.7%	85	59.9%	0.4
**CG**	27	38.0%	45	31.7%	
**GG**	3	4.2%	12	8.5%	
**rs 9505118**					
**Alele**					
**A**	89	62.6%	164	57.7%	0.3
**G**	53	37.3%	120	42.2%	
**Genotype**					
**AA**	23	32.4%	44	31.0%	0.3
**AG**	42	59.2%	76	53.5%	
**GG**	6	8.5%	22	15.5%	
**rs10811661**					
**Alele**					
**C**	29	20.4%	53	18.6%	0.6
**T**	113	79.5%	231	81.3%	
**Genotype**					
**CC**	4	5.6%	8	5.6%	0.6
**CT**	23	32.4%	38	26.8%	
**TT**	44	62.0%	96	67.6%	

The rs 266729 polymorphism was in Hardy–Weinberg equilibrium (HWE) in GDM cases (*p* = 0.57), and control cases (*p* = 0.09). The rs 9505118 polymorphism was out of HWE in GDM cases (*p* = 0.03), but in control cases, it was in HWE (*p* = 0.24). The rs10811661 polymorphism was in HWE in GDM cases (*p* = 0.66), and control cases (*p* = 0.11).

**Table 6:** Correlations between rs266729, rs10811661, and rs9505118 polymorphisms and maternal-fetal outcomes in pregnant women with GDM (*n* = 71).

Variables/Genotypes	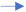	rs266729-CG+GG vs. CC	rs266729-CG+CC vs. GG	rs9505118-AA+AG vs. GG	rs9505118-GG+AG vs. AA	rs10811661-CT+TT vs. CC	rs10811661-TT vs. CC+CT
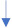							
**Preterm birth**	r	−0.124	0.058	0.084	0.073	−0.171	0.011
	*p*-value	0.303	0.632	0.488	0.546	0.153	0.926
**Labor induction failure**	r	0.246 *	0.044	−0.188	−0.154	0.051	−0.165
	*p*-value	0.039	0.715	0.117	0.200	0.671	0.170
**Perineal lacerations**	r	−0.034	−0.147	−0.052	0.151	0.087	−0.279 *
	*p*-value	0.776	0.223	0.667	0.207	0.470	0.018
**Macrosomia (≥4000 g)**	r	−0.093	−0.063	−0.215	0.063	0.126	0.092
	*p*-value	0.438	0.603	0.072	0.600	0.293	0.445
**Gestational hypertension**	r	0.071	0.095	−0.133	0.152	−0.053	0.189
	*p*-value	0.558	0.432	0.268	0.207	0.662	0.115

*. Correlation is significant at the 0.05 level (2-tailed). Spearman correlation.

## Data Availability

The data that support the findings of this study are available from the corresponding author C.M. upon reasonable request.
